# Oral Health Problems among Canadians Aged 45 to 85: Data from the Canadian Longitudinal Study on Aging Baseline Survey (2011–2015)

**DOI:** 10.3390/ijerph20085533

**Published:** 2023-04-17

**Authors:** Vanessa De Rubeis, Ying Jiang, Margaret de Groh, Lisette Dufour, Annie Bronsard, Howard Morrison, Fahad Butt, Carol Walker Bassim

**Affiliations:** 1 Department of Health Research Methods, Evidence, and Impact, McMaster University, Hamilton, ON L8S 4L8, Canada; 2 Applied Research Division, Centre for Surveillance and Applied Research, Public Health Agency of Canada, Ottawa, ON K0A 0K9, Canada; 3 Office of the Chief Dental Officer, Public Health Agency of Canada, Ottawa, ON K0A 0K9, Canada; 4 Health Sciences, Department of Medicine, McMaster University, Hamilton, ON L8S 4L8, Canada

**Keywords:** oral health, dentate and edentate, CLSA

## Abstract

Oral health is a critical component of overall health. The objective of this study was to describe oral health problems among 47,581 adults aged 45 to 85 in the Canadian Longitudinal Study on Aging (CLSA) among those who have at least one natural tooth (92%) and those without natural teeth across various demographic categories. Among the 47,581 participants in the study, 92% reported having at least one natural tooth (dentate). Among those without teeth, 63% reported an income less than CAD 50,000 versus 39% among those with teeth. Whether they had teeth or not, over 30% of people reported two or more oral health problems. Older adults appear to be retaining their natural teeth (28.9%), but still report experiencing oral health problems. As the population ages, loss of all teeth may not be the most useful proxy for poor oral health, and a population-level understanding of oral health problems may help to better define poor oral health.

## 1. Introduction

Oral health is an important component of overall health, well-being, and quality of life [1]. Globally, oral health problems still remain a significant problem to public health, with dental caries and periodontal disease affecting a significant proportion of the population from children to older adults [2]. However, the burden of oral disease typically has the highest prevalence among the oldest age groups [3]. Specifically, in Canada, 14.2% of adults aged 60–79 years have reported having poor oral health, which is particularly concerning given the aging population [1]. The proportion of seniors aged 65 and older is expected to grow by 70% in the next 20 years [4]; accordingly, the prevalence of oral health-related problems is also expected to increase. 

Oral health problems include more than dental caries, gum disease, tooth loss, or oral cancer [5]. It also includes problems with dentures, difficulties eating, or problems associated with the mouth or jaw [1]. Oral health problems have been found to be associated with poor self-perceived general health, and with various chronic conditions, particularly among individuals of lower socioeconomic status [6,7]. Given the aging population, and the association between oral health and perceived overall general health, it is important to explore potential factors associated with oral health to allow for better adapted interventions to be developed. Additionally, problems related to oral health may be prevented; however, without understanding the prevalence of these problems at a national-level, targeted prevention strategies cannot be developed, and guidance cannot be made at the policy or practice level. 

A previous study conducted using the Canadian Longitudinal Study on Aging (CLSA) found that self-reported oral health and dental service use varied by several different socioeconomic and demographic factors and regions across Canada. For instance, the highest prevalence of fair/poor oral health was reported by individuals residing in New Brunswick (9.9%), followed by Alberta (9.2%) [6]. This study also noted increased self-reported poor oral health by people of lower income status, as well as those who smoked, were depressed or had poor self-rated general health [6]. However, this study did not specifically look at the different problems that oral health is composed of, including teeth problems (e.g., loose or broken teeth), denture problems (e.g., broken or missing dentures) and gum problems (e.g., sore or bleeding gums).

One factor that is indicative of poor oral health is edentulism, which is the complete loss of natural teeth [6,8]. Older adults are the most likely to suffer from edentulism, with 15% of Canadian adults aged 65 years and older found to be edentate [6]. Edentate adults are more likely to report oral health problems: for instance, the Canadian Health Measure Survey found almost 26% of Canadian adults who were edentulous reported they avoided food because of problems with their mouth [1]. However, it is becoming more common for older adults to retain their natural teeth, suggesting the importance of understanding predictors of oral health among people with and without natural teeth [1]. The objective of this study was to describe the prevalence of oral health problems among adults aged 45 to 85 in the CLSA by dentate and edentate status. Differences in the prevalence of oral health problems were also explored by sex. 

## 2. Methods

The Strengthening the Reporting of Observational Studies in Epidemiology (STROBE) Statement for cross-sectional studies guided the preparation of this manuscript [9].

### 2.1. Study Design, Settingdata Source, and Participants

**Figure 1:**
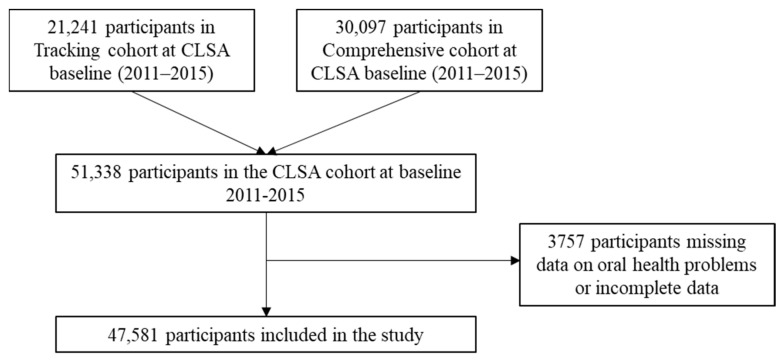
Participant flow diagram of Canadian Longitudinal Study on Aging (CLSA) participants included in the current study at baseline (2011–2015).

A cross-sectional study was conducted using secondary data analysis of the CLSA baseline (2011–2015) data. The CLSA is a longitudinal cohort study following 51,338 individuals aged 45 to 85 years at the time of recruitment for over 20 years. To be eligible for inclusion into the CLSA, participants had to reside in one of the 10 Canadian provinces, complete the questionnaires in English or French, have the cognitive ability to participate on their own, and they not be part of the Canadian Armed forces or live on a First Nations reserve. A detailed methodology of the CLSA has previously been published [10]. A total of 3757 people were removed who were missing data on oral health, or incorrectly reported oral health problems (e.g., people who reported having problems with their teeth but also reported not having teeth), leaving a final sample of 47,581 (Figure 1). Ethics approval for this study was received from the Public Health Agency of Canada/Health Canada Research Ethics Board on 24 June 2019 (project id: 2014–0015).

### 2.2. Oral Health

Self-perceived oral health was measured using the question, “In general, would you say the health of your mouth is excellent, very good, good, fair, or poor?” Responses were dichotomized as poor/fair and good/very good/excellent. Oral health was also measured by asking participants if they had experienced oral health problems in the past 12 months, including toothache; an inability to chew adequately; loose/ill-fitting, broken, or missing dentures; swelling in the mouth; dry mouth; burning mouth; sore jaw muscles; painful jaw joints; natural tooth decay; natural loose tooth; gums around natural teeth being sore; gums around natural teeth bleeding; denture-related sores; dirty teeth or dentures; and bad breath. Some oral health problems were grouped together given the small proportion of people who reported them (Table 1). To measure if participants had natural teeth, they were asked “Do you have one or more of your original teeth?”. If participants answered yes, then they were classified as dentate; if they answered no, they were classified as edentate, indicating they had no natural teeth. Participants were also asked, “Do you wear dentures or false teeth?”. People among the dentate group were further classified as wearing dentures versus no dentures. Stratification by denture status was not performed for edentate participants given the small sample size.

### 2.3. Other Covariates

Potential confounding variables included age (categorized as 45–54, 55–64, 65–74, 75–85), sex (male or female), total household income (<CAD 50,000, CAD 50,000–CAD 100,000, >CAD 100,000) and education (categorized as secondary school or less versus post-secondary degree/diploma). Self-perceived general health was measured using the question, “In general, would you say your health is excellent, very good, good, fair, or poor”. Response options ranged from poor to excellent. Since there was not an equal distribution of responses to all 5 options, perceived general health were dichotomized into poor/fair vs. good/very good/excellent. Poor general health was operationally defined by participants reporting the presence of at least one chronic condition including, but not limited to, diabetes, cancer, heart disease, Alzheimer’s, Parkinson’s, allergies. (Yes ≥ 1 conditions vs. No = 0 conditions). This classification of chronic conditions has previously been used in other Canadian population surveys [11].

### 2.4. Statistical Analysis

Statistical analyses were completed using SAS 9.4. This study was conducted following a descriptive epidemiology framework [12]. The prevalence of oral health problems was estimated using weighted frequencies which were calculated as percentages using sampling weights, and chi-square tests were used to estimate if groups were statistically different. The prevalence of oral health problems was stratified by important population descriptors including edentate status and sex.

## 3. Results

Among the sample (n = 47,581), 92% of people reported having one or more natural teeth (dentate), and the remaining 8% reported having no natural teeth (Table 1). The majority of those with teeth (71%) were aged 45 to 64 years, whereas the majority of those without teeth (61%) were 65 to 85 years. A high proportion of people without teeth (63.0%) reported a total household income less than CAD 50,000. It was determined that 81.1% of people within this household income group were less than 65 years of age, suggesting retirement was not the driving factor behind the lower reported household income (data not shown). Regardless of edentulism status, over 90% of respondents perceived their oral health as good, very good or excellent, even though 26.6% of dentate and 34.7% of edentate respondents reported two or more oral health problems. 

Problems with dentures, including denture-related sores, uncomfortable dentures, and dentures being loose or not fitting were reported by a higher proportion of people within the edentate group. Problems with gums were reported by more people with teeth compared to those without teeth across both males and females. A complete description of oral health problems among males and females by edentulism status can be found in Table 2.

## 4. Discussion

We found that different demographic characteristics increase the risk of edentulism, while a high proportion of all participants report experiencing at least one oral health problem. Although most (60%) reported having one or more oral health problems, 93% of the dentate sample and 91% of the edentate sample perceived their oral health as good, very good, or excellent. We did not find differences when reporting oral health problems among males compared to females. 

Given the established association between oral health and general health, the focus is often put on preventing oral health problems to support improving overall health [6,7,13]. As people age, the burden of disease associated with oral health increases [1]. Edentulism status is often used as a proxy for determining the oral health of a population [1,8]. However, during the last decade, it has been shown that older adults are retaining more natural teeth as they age, but are still experiencing oral health problems, which is consistent with the findings of this study [14]. Given the aging population, and the increase in oral disease among older adults, it is critical that prevention and intervention strategies are developed to reduce the burden associated with oral health problems. Self-perceived oral health also does not appear to be a perfect indicator of oral health, as we found over 90% of people among both the dentate and edentate groups perceived their oral health as good, very good, or excellent, but still reported experiencing oral health problems. It is also important to note the estimate of self-perceived good, very good, or excellent oral health reported in the current study is slightly higher than a previous estimate from the Canadian Health Measures Survey (CHMS) back in 2007–2009, which found 84.5% of Canadians reported their oral health to be good, very good, or excellent [1]. 

Oral health problems are prevalent globally [15]. It is estimated that oral health problems affect 3.5 billion people around the world. The global prevalence of edentulism for adults is estimated to be about 7%, with 23% of those 60 years old and older being edentulous [5]. Our findings for Canadians older than 45 years old was that 8% of people have reported having no natural teeth, with about 12% of those aged 65–74 years and about 20% 75 years and older being edentulous. Older Canadians, similar to the patterns of other economically developed countries [15], are retaining teeth in older age [16]. In our study, those with teeth and those edentulous continue to express that they have oral health problems across all age groups. 

Similar to global reports, the most commonly reported oral health problems in our study were problems with gums and tooth decay among both males and females [17]. It is estimated that almost 2.5 billion and 1 billion people are affected by tooth decay and severe gum disease, respectively, across the world [17]. Differences have been noted in the prevalence of oral health problems by demographic characteristics, such as age, education, race or income [18,19]. Although we explored edentulism status by different socioeconomic factors, we did not explore variation in oral health problems, highlighting the need for continued research. Clinical examinations by a dentist during the CHMS found that more than one-third of the participants (34% of dentate and 41% of edentate) needed dental treatment [16]. Self-rated oral health is associated with oral health indicators such as clinical measures of caries, or loss of periodontal attachment [20]. The current study captures this oral health burden reflecting oral disease, as well as expanding into a greater definition of oral health comprising denture comfort, chewing ability, and dry mouth. 

### Strengths and Limitations

This study contributes to the literature on understanding oral health problems among the aging population within Canada using the CLSA and its large sample size of over 47,000 people. The CLSA is a novel data source that has collected comprehensive information on oral health problems, as well as other demographic and behavior-based information. The study applied sampling weights, allowing for better inferences to be made and overcoming biases associated with how participants were recruited. 

The limitations of this current study include its cross-sectional design, which makes it difficult to establish temporality. However, with the future availability of CLSA follow-up data, this limitation can be overcome, as longitudinal studies can be conducted. Furthermore, data were self-reported; previous studies have noted potential inaccuracies associated with self-reported recall [21,22]. These differences in the ability to self-report oral health problems may be related to the specific oral health problem, or different socioeconomic or demographic factors [21] Finally, it is important to note that the CLSA sample may not be representative of the Canadian population; however, the findings of this paper can be generalized to people who share similar characteristics to those who are included in the current study [23].

Although this paper utilized a descriptive approach, it is helpful for understanding the burden of oral health problems among Canadian adults. The findings of this research can be used to inform research questions that plan to use an analytic approach to study risk factors for oral health problems. These findings can be used by public health professionals who are interested in developing strategies to improve oral health, thus improving overall health and well-being. For instance, these findings provide evidence that various oral health problems are prevalent among Canadian adults, and this varies by edentulism status, which can be used to design potential programming that may be used to promote healthy behaviors that could also, in turn, promote better oral health. Finally, oral health professionals can use the findings from this paper to help to identify those who are at high risk of oral health problems, and therefore provide them with education regarding the importance of oral health.

## 5. Conclusions

The findings of this study suggest that edentulism status may not be the best proxy for understanding the burden of disease associated with oral health, as we found that the proportion of people reporting oral health problems was comparable regardless of edentulism status. It was also apparent that self-perception of oral health was not a good indicator of oral health problems. Identifying high-risk subgroups within the population who may be at a greater risk of increased oral health problems, and a better understanding of what those oral health problems are, could be used to inform prevention strategies. 

## Figures and Tables

**Table 1:** Characteristics of Canadian adults from the Canadian Longitudinal Study on Aging at baseline (2011–2015), by teeth status.

Variables	Dentate (with Teeth)				Edentate (without Teeth)
	Overall	No Dentures	With Dentures	*p*-Value ^1^	
Age group 45–54 55–64 65–74 75–85	39.8% 31.3% 18.4% 10.5%	45.2% 31.1% 15.9% 7.8%	21.1% 32.1% 26.9% 19.9%	<0.0001	11.4% 27.1% 31.1% 30.3%
Sex Male Female	48.9% 51.1%	49.1% 50.9%	48.2% 51.8%	0.30	44.0% 56.0%
Household income <CAD 50,000 CAD 50,000–100,000 >CAD 100,000	24.5% 37.2% 38.4%	20.3% 36.3% 43.4%	39.0% 40.2% 20.9%	<0.0001	63.0% 27.0% 10.0%
Education Secondary school or less Post-secondary degree/diploma	24.5% 75.5%	21.7% 78.3%	34.3% 65.7%	<0.0001	50.6% 49.4%
Chronic conditions No At least 1	10.7% 89.3%	11.7% 88.3%	7.0% 93.0%	<0.0001	4.5% 95.5%
Self-perceived general health Poor/fair Good/very good/excellent	10.0% 90.0%	9.1% 90.9%	13.1% 86.9%	<0.0001	20.0% 80.0%
Self-perceived oral health Poor/fair Good/very good/excellent	7.3% 92.7%	6.0% 94.0%	11.9% 88.1%	<0.0001	8.7% 91.3%
Number of oral health problems 0 1 2 or more	38.0% 25.3% 26.6%	39.3% 25.8% 34.9%	33.7% 23.8% 42.5%	<0.0001	38.8% 26.5% 34.7%

1. Chi-square test performed to determine if people among the dentate group who had dentures were statistically different than those without dentures.

**Table 2:** Percentage of oral health problems among males and females from the Canadian Longitudinal Study on Aging (CLSA) at CLSA baseline (2011–2015), by teeth status.

Oral Health Problems	Males					Females				
	Dentate (with teeth)				Edentate (without Teeth)	Dentate (with Teeth)				Edentate (without Teeth)
	Overall	No Dentures	Dentures	*p*-Value ^1^		Overall	No Dentures	Dentures	*p*-Value ^1^	
Oral Health Problems No 1 2 or more	38.3% 25.3% 36.4%	39.2% 26.1% 34.7%	35.4% 22.5% 42.1%	<0.0001	43.4% 24.8% 31.8%	37.8% 25.4% 36.8%	39.4% 25.5% 35.1%	32.2% 25.0% 42.8%	<0.0001	35.2% 27.8% 37.0%
Teeth Problems										
Tooth Loose	5.7%	4.9%	8.5%	<0.0001	N/A	6.2%	4.7%	11.0%	<0.0001	N/A
Tooth Broken	14.5%	15.2%	12.1%	0.001	N/A	11.3%	11.9%	9.2%	0.001	N/A
Toothache	16.6%	17.1%	14.8%	0.02	N/A	14.0%	14.3%	13.1%	0.18	N/A
Tooth Decay	20.1%	20.2%	19.8%	0.70	N/A	16.0%	15.6%	17.3%	0.08	N/A
Denture Problems										
Denture-related sores, uncomfortable, loose/do not fit ^2^	4.4%	N/A	19.9%	N/A	34.3%	4.4%	N/A	19.2%	N/A	38.1%
Dentures broken/missing ^2^	1.2%	N/A	5.4%	N/A	5.1%	1.1%	N/A	4.6%	N/A	3.1%
Gum Problems										
Gums sore/bleed	21.7%	22.5%	19.0%	0.001	2.3%	20.4%	20.8%	18.8%	0.06	2.3%
Other Oral Health Problems										
Swelling/burning in mouth ^2^	6.2%	5.7%	7.8%	0.002	7.9%	7.4%	6.7%	9.3%	0.0002	9.0%
Jaw muscles sore/joints painful ^2^	6.8%	7.0%	5.7%	0.05	5.6%	12.6%	13.5%	9.6%	<0.001	10.2%
Cannot chew adequately	8.7%	8.1%	11.2%	<0.0001	12.3%	9.4%	8.4%	12.7%	<0.0001	17.6%
Dry mouth	15.8%	15.1%	18.6%	0.0002	23.6%	19.3%	17.6%	24.7%	<0.0001	31.8%
Teeth or dentures dirty, bad breath, and others ^2^	15.4%	16.1%	12.6%	0.0002	10.6%	13.2%	13.1%	13.6%	0.53	11.4%

1. Chi-square test performed to determine if people among the dentate group who had dentures were statistically different than those without dentures. 2. Oral health problems collapsed due to small sample sizes. Participants reported one or more of listed problems

## Data Availability

Data are available from the Canadian Longitudinal Study on Aging (www.clsa-elcv.ca for researchers who meet the criteria for access to de-identified CLSA data.
